# MiR-197 Inhibitor Loaded AbCD133@MSNs@GNR Affects the Development of Prostate Cancer Through Targeting ITGAV

**DOI:** 10.3389/fcell.2021.646884

**Published:** 2021-06-14

**Authors:** Guanqun Ju, Yingjian Zhu, Tao Du, Wanli Cao, Jianhai Lin, Chun Li, Dongliang Xu, Zhijun Wang

**Affiliations:** ^1^Department of Urology, Changzheng Hospital, Naval Medical University, Shanghai, China; ^2^Department of Urology, Shanghai Jiangqiao Hospital, Shanghai General Hospital Jiading Branch, Shanghai, China; ^3^Department of Urology, Henan Provincial People’s Hospital, Zhengzhou, China; ^4^Key Laboratory of Functional Genomic and Molecular Diagnosis of Gansu Province, Lanzhou, China; ^5^Urology Centre, Shuguang Hospital Affiliated to Shanghai University of Traditional Chinese Medicine, Shanghai, China

**Keywords:** prostate cancer, cancer stem cell, gold nanorod, miR-197, ITGAV

## Abstract

Prostate cancer is one of the most severe male malignant tumors, which ranks second in mortality rate among all tumors. Traditional methods of treatment for prostate cancer produce obvious side effects and a high recurrence rate. Cancer stem cells are considered to be a group of cells that determine the proliferation, metastasis, and drug resistance of tumor. Prostate cancer therapy based on microRNAs and prostate cancer stem cells (PCSCs) has been a research hot spot in this field. Previous studies have reported that miR-197 plays an important role in the occurrence and development of prostate cancer, but the molecular mechanism of miR-197 on the development of prostate cancer has not been reported yet. In this study, we verified that miR-197 is significantly overexpressed in prostate cancer tissues and prostate cancer cells. Then, we verified that miR-197 expression affects the proliferation, invasion, and metastasis of prostate cancer cells by regulating integrin subunit alpha V (ITGAV) expression through STAT5 pathway, and the results indicated that the miR-197 inhibitor can be a prostate cancer suppressor. Then we synthesized the AbCD133@GNR@MSNs@miR-197 inhibitor drug carrier, in which 35.42 μg of the miR-197 inhibitor could be loaded in 1 mg of AbCD133@GNR@MSNs. The AbCD133@GNR@MSNs@miR-197 inhibitor demonstrated good photothermal properties and photothermal controlled-release properties. The modified CD133 antibodies on the surface of the nano drug carrier helped more drug carriers to enter the PCSCs. The pharmacodynamic effects of the AbCD133@GNR@MSNs@miR-197 inhibitor on PCSCs *in vivo* and *in vitro* were studied under near-infrared radiation. The results showed that the AbCD133@GNR@MSNs@miR-197 inhibitor prepared in this study could not only significantly suppress the development of PCSCs through ITGAV/STAT5 pathway but also significantly suppress the growth of PCSC solid tumors. In short, our study verified that miR-197 regulates the development of PCSCs through STAT5 pathway by targeting ITGAV, and the AbCD133@MSNs@GNR@miR-197 inhibitor could be a potential suppressor used in prostate cancer treatment. In short, our study found that miR-197 affected the development of prostate cancer by regulating ITGAV. The AbCD133@GNR@MSNs@miR-197 inhibitor prepared in this study could suppress the development and growth of PCSCs *in vitro* and in solid tumors not only by targeting the ITGAV but also through photothermal therapy. Our study not only provides a theoretical basis for the clinical treatment of prostate cancer but also provides a research scheme of drug loading and microRNA-based photothermal controlled therapy for prostate cancer.

## Introduction

Prostate cancer (PC) is a major malignancy that occurs in middle-aged and elderly men, with an incidence rate of 25.3/10 million worldwide. It is also the second most common cancer worldwide and the fifth leading cause of cancer death. In 2018 alone, there were nearly 1,300,000 PC cases and 359,000 new deaths as a result of PC (Aggarwal et al., 2015; Grozescu and Popa, 2017; Bray et al., 2018; Taneja, 2020). PC is usually asymptomatic during the early stage, and most patients are diagnosed during the middle and late stages. Endocrine therapy is the first choice of treatment for advanced PC. However, endocrine therapy is not effective for advanced metastatic PC or castration-resistant PC patients (Shao et al., 2012). At present, the exact molecular mechanism by which PC develops and progresses is not clear. Therefore, it is of great significance to study the molecular mechanisms associated with the occurrence and development of PC and develop new targeted drug carriers for the treatment of PC.

Cancer stem cells (CSCs) are a small number of cells that drive the occurrence and development of tumor. CSCs can maintain the stem cell characteristics of tumor cell growth and can differentiate into the whole heterogeneous tumor (Bonnet and Dick, 1997; Gratwohl et al., 2002; Orkin and Zon, 2008; O’Connor et al., 2014). Prostate CSCs (PCSCs) were first reported in 2007 (Miki et al., 2007). Studies have shown that PCSCs have self-renewal and unique tumorigenicity *in vivo* and that PCSCs determine the development of PC; in addition, the resistance of PC to conventional chemotherapy and radiotherapy may be partly due to the existence of PCSCs (Hermann et al., 2007; Huang et al., 2021; Navarro-Marchal et al., 2021; Zhang et al., 2021). Therefore, research and development of drugs targeting PCSCs is one of the important ways to treat PC.

MicroRNAs (miRNAs) are a type of endogenous non-coding RNAs found in eukaryotic cells, with a length of 18–25 nucleotides. MiRNAs can bind to the 3′ untranslated region of target gene mRNA and can regulate the expression of downstream target genes by changing the stability of the mRNA. In recent years, abnormal levels of miRNA expression have been found in malignant tumors, which have been found to be closely associated with the proliferation, differentiation, and apoptosis of tumor cells (Dimakakos et al., 2014; Brzeszczyńska et al., 2020; Paul et al., 2020). Studies have shown that the level of miR-197 expression in the serum of patients with PC is higher than that of benign prostatic hyperplasia patients and healthy males, and the difference is statistically significant (Yuan et al., 2019). These results suggest that miR-197 plays an important role in the occurrence and development of PC. Therefore, miR-197-related drugs could potentially be used to treat PC bone metastasis. However, the molecular mechanism by which miR-197 exerts its function in bone metastasis of PC has not yet been reported on. MiR-197 would need to efficiently enter cells for it to be used for the treatment of PC. In addition, there are various degradation mechanisms that lead to the degradation of miR-197 in cells. At present, the clinical application of miR-197 lacks efficiency, accuracy, and a targeted controlled-release delivery vector. Research conducted on tumor drug targeting carriers has found that the targeting property of a targeting agent is associated with the targeting performance of the whole carrier, which is a key factor for tumor targeting (Kaur et al., 2020; Prasher et al., 2020; Schneider-Rauber et al., 2020). Therefore, it is necessary to identify drug delivery carriers with high transfection efficiency, good targeting performance, and controlled release. Mesoporous silica nanoparticles (MSNs) provide many advantages, including a simple process for synthesis, easy surface modification, and good biocompatibility. MSNs have become an inorganic carrier material that has been widely studied in the field of drug carriers during recent years (Hargrove et al., 2020; Taweekarn et al., 2020; Wu et al., 2020). Gold nanorods (GNRs) are photothermal materials that can generate heat after near-infrared laser response. The surfactant cetyltrimethylammonium bromide (CTAB) formed on the surface of solid GNRs has high cytotoxicity, and it cannot load drugs. Coating the surface of GNRs with mesoporous silica can not only reduce the toxicity of the material but also make use of the high specific surface area that mesoporous silica will have for drug loading. GNRs can melt phase change materials (PCMs), which block mesoporous silica after heating through near-infrared laser irradiation, so as to achieve photothermal controlled release of drugs (Das et al., 2020; Khelfa et al., 2020; Lebepe et al., 2020).

In this study, we first studied the expression of miR-197 in PC tissues and PC cells. Then, we predicted and verified the relationship between miR-197 and the target protein, integrin subunit alpha V (ITGAV), through a bioinformatics analysis and double luciferase experiments. Further studies were performed to study the molecular mechanism by which miR-197 regulates the occurrence and development of PC by regulating the expression of ITGAV. Subsequently, we synthesized MSN-coated GNRs, GNR@MSNs, which were loaded with the miR-197 inhibitor. Then, the mesoporous GNR@MSNs were sealed using PCMs to create the GNR@MSNs@miR-197 inhibitor. The AbCD133@GNR@MSNs@miR-197 inhibitor was synthesized by modifying the CD133 antibody on the surface of the GNR@MSNs@miR-197 inhibitor. Thereafter, we studied the pharmacodynamic effect of the AbCD133@GNR@MSNs@miR-197 inhibitor on PC *in vivo* and *in vitro* under near-infrared radiation. Overall, our study provides a theoretical basis for the clinical treatment of PC.

## Materials and Methods

### Cell Culture

The DU145, PC3, and LNCaP cell lines were obtained from the Cell Bank of the Institute of Biochemistry and Cell Biology (Shanghai, China). RPMI-1640 (Thermo Fisher Scientific, Inc., Waltham, MA, United States) culture medium was used for the DU145, PC3, LNCaP, and PCSCs, while the culture medium used for RWPE-1 was supplied by Hunan Fenghui Biotechnology Co., Ltd. All media were supplemented with 10% fetal bovine serum (FBS; Thermo Fisher Scientific, Inc.) and were kept at 37°C in a humidified 5% CO_2_ incubator.

### Prostate Cancer Stem Cell Screening

PC3 cells of 10^8^ were washed with phosphate-buffered saline (PBS) three times; then cells were incubated with fluorescein isothiocyanate (FITC)-labeled CD133 antibody in the dark; the cells were collected with centrifugation at 1,000 rpm for 5 min and then washed with PBS two times; and the PCSCs was screened from PC3 cell by use of Aria SORP flow cytometry (BD, United States).

### Cell Transfection

Human miR-197 mimics and negative control oligonucleotides (NCOs) were purchased from Beenbio Co., Ltd. (Shanghai, China). Cells were seeded into six-well plates at a density of 4 × 10^5^ cells/well for plasmid transfection. Subsequently, these RNA oligonucleotides or plasmids were transfected into cells using a Lipofectamine^®^3000 system (Thermo Fisher Scientific, Inc.) by following the manufacturer’s instructions.

### Quantitative Real-Time Polymerase Chain Reaction

Total RNA was extracted using Trizol reagent (Thermo Fisher Scientific, Inc.). To obtain the cDNA, the extracted RNAs were reverse transcribed using a One Step PrimeScript miRNA cDNA Synthesis Kit (TaKaRa, Dalian, China), according to the manufacturer’s instructions. qPCR was performed following standard protocol using a SYBR Green PCR kit (Toyobo, Japan). The following qPCR primer was used for miR-197: forward 5′-GTTCACCACCTTCTCCAC-3′; reverse 5′-GTGCAGGGTCCGAGGT-3′. U6 small nuclear RNA (snRNA U6) was used as the internal reference to determine the relative expression of miR-197 through the 2^–ΔΔ*CT*^ method.

### MTT Assay

Cells were seeded onto a 96-well plate with 100 μl of culture medium at a density of 5,000 cells/well. The cells were incubated at 37°C in a humid atmosphere with 5% CO_2_ for 24 h. At the end of the 48-h, 10 μl of MTT reagent obtained from Beenbio Co., Ltd. (Shanghai, China) was added to the culture medium for 4 h of incubation. The cells were then suspended, and absorbance was detected at 570 nm using an enzyme-linked immunosorbent assay microplate reader (Sunrise Microplate Reader; TECAN, Männedorf, Switzerland).

### Luciferase Reporter Assay

ITGAV 3′-UTR fragments containing the predicted miR-197 binding site were amplified using the cDNA of the PC3 cell. Subsequently, the fragments were inserted into pGL3 luciferase reporter vectors (Promega, Madison, WI, United States). For the luciferase reporter assay, PC3 cells were co-transfected with the pGL3 reporter and ITGAV UTR together with either NCO or miR-197 mimics using a Lipofectamine^®^3000 system. Twenty-four hours after transfection, the cells were collected and lysed. Then, the luciferase activity was determined using a dual-luciferase reporter assay system (Promega), according to the manufacturer’s instructions.

### Western Blotting Analysis

The treated cells were harvested and incubated in radioimmunoprecipitation assay (RIPA) lysis buffer (Cell Signaling Technology Inc., Danvers, MA, United States) for 30 min at 4°C to isolate total proteins. The extracted proteins were then separated using 10% sodium dodecyl sulfate–polyacrylamide gel electrophoresis (SDS-PAGE) and transferred onto a polyvinylidene fluoride membrane (Roche, Basel, Switzerland). Subsequently, the membranes were incubated with primary antibodies (Santa Cruz Biotechnology, Santa Cruz, CA, United States) at 4°C overnight and then incubated with a secondary antibody (Santa Cruz Biotechnology) at room temperature for 2 h. The protein bands were visualized using an enhanced chemiluminescence (ECL) western blotting substrate (Thermo Fisher Scientific, Inc.).

### Flow Cytometry Analysis

Cell apoptosis was detected using flow cytometry analysis. Annexin V-FITC and propidium iodide (PI) (BD Pharmingen, San Diego, CA, United States) were used to calculate the number of Annexin V-positive cells.

### Gold Nanorod Preparation

First, 5 ml of 0.5 mM HAuCl_4_ solution was mixed with 5 ml of 0.2 M CTAB. Then, 0.6 ml of frozen 0.01 M NaBH4 was added, and the mixture was stirred for 2 min and allowed to stand for 2 h at 25°C. Thereafter, 5 ml of 0.2 M CTAB, 5 ml of 1 mM HAuCl_4_, 0.5 ml of AgNO_3_, and 0.07 ml of 0.1 M acetic acid (AA) were added into the reaction vessel and stirred for 2 min. Finally, 0.012 ml of the seed solution was added into the growth solution, stirred for 2 min, and allowed to stand at 28°C for 3 h to obtain fully grown GNRs.

### GNR@MSN Preparation

The prepared GNRs were washed twice with PBS, and excess CTAB was removed through centrifugation and then redistributed in 40 ml of PBS. Then, 50 μl of ammonia (25, wt%) was added to adjust the GNR aqueous solution to pH 10, and then 0.5 ml of 10 mM TEOS/ethanol solution was added at the rate of 3.5 ml/h. The reaction was carried out at 40°C for 24 h. The reaction products were centrifuged several times with ethanol and water. To remove the CTAB residual molecules from the mesoporous channels, 60 ml of ethanol/ammonium nitrate solution (10 mg/ml) was added to reflux for 6 h using the ion exchange method. Thereafter, the solution was centrifuged with ethanol for purification. Finally, the GNR@MSN products were dispersed in deionized water for preservation.

### Characterization

The nitrogen adsorption–desorption curve was created using a micrometrics Tristar 3000 system at 77 K. The sample was pre-dehydrated at 120°C for 6 h before determination. The specific surface area and pore volume were calculated using the Brunauer–Emmett–Teller (BET) formula and Barrett–Joyner–Halenda (BJH) model, respectively. The transmission electron microscopy (TEM) image was measured using a jem-2010f-hr electron microscope at a voltage of 200 kV. During sample preparation, the sample was first refined, dispersed in ethanol solution, and then ultrasonically treated. Then, the suspension solution was dropped onto a copper mesh coated with carbon film and was placed on the sample table for vacuumization and observation.

### Detection of Heat Production in the GNR@MSNs Under 808-nm Laser Irradiation

The diluted GNR@MSN solution was irradiated with an 808-nm near-infrared laser for different durations and at different intensities (spot size: 5 mm). The temperature of the solution was measured using an infrared temperature detector.

### Fourier-Transform Infrared Analysis of the GNR@MSNs

GNR@MSNs were mixed with KBr powder, grinded to a uniform size, and pressed onto a 1-mm-thick transparent sheet. Fourier-transform infrared (FTIR) analysis was performed using a nexus-470 infrared instrument.

### MiR-197 Inhibitor Loading

Since the melting point of PCM tetradecanol is 38–39°C, a sufficient amount of a miR-197 inhibitor and PCM needs to be mixed for loading onto the GNRs@MSNs at a temperature higher than the melting point. First, 100 mg of tetradecanol was heated at 60°C until it had melted fully. Then, 20 μg of the miR-197 inhibitor was added and thoroughly mixed through magnetic stirring for 2 h. Then, 100 mg of the GNRs@MSNs was mixed with a large amount of chloroform, added into the PCM mixture solution, and stirred for 2 h at 60°C. During this mixing process, through the volatilization of trichloromethane, the PCM@miR-197 inhibitor mixture slowly infiltrated the mesopore cavities of the GNRs@MSNs. Finally, an excess amount of hot water was added to the abovementioned preparation system to produce two phases that were obviously incompatible with each other: the GNRs@MSN water phase with the PCM@miR-197 inhibitor and the chloroform phase without the PCM@miR-197 inhibitor encapsulation. The two phases were rapidly separated, and the water phase was immersed in an ice water solution for cooling for 1 min and then centrifuged for 5 min at 12,000 rpm. The precipitates were washed eight times with precooled deionized water and then freeze-dried for 24 h at −50°C and 0.8 mbar in a vacuum. The GNRs@MSNs loaded with the PCM@miR-197 inhibitor were named the GNRs@MSNs@PCM@miR-197 inhibitor. In addition, with the use of the same preparation method, the molecules constructed that did not contain the miR-197 inhibitor were named GNRs@MSNs@PCM.

### Testing the Release Capacity of GNRs@MSNs@PCM@miR-197 Inhibitor

To test the miR-197 inhibitor release property of GNRs@MSNs@PCM with or without the effect of an 808-nm laser, the GNRs@MSNs@PCM@miR-197 inhibitor was dispersed in 5 ml of PBS (pH 7.4) in semipermeable dialysis bags at 37°C by gentle shaking. Then, 1 ml of the released medium was removed, and 1 ml of fresh medium was added. The amount of the miR-197 inhibitor released was measured using a NanoDrop 3000 spectrophotometer.

### Cellular Uptake and Internalization

A total of 2 × 10^4^ of PCSCs were seeded into glass-bottom plates (35 mm, Corning Incorporated) in Dulbecco’s modified Eagle’s medium (DMEM) with 10% FBS and were incubated at a final concentration of 50 μg/ml of FITC-labeled GNRs@MSNs@PCM and FITC-labeled AbCD133@GNRs@MSNs@PCM for 24 h. The cells were incubated with 60 nM of LysoTracker Red DND-99 (Beyotime Institute of Biotechnology, Haimen, China) for 1 h at 37°C. After being washed with PBS, the cells were fixed with 4% paraformaldehyde and stained with 10 μg/ml of 4′,6-diamidino-2-phenylindole (DAPI; Sigma). The cells were then washed three times with PBS and mounted. The micrographs were first observed under a Nikon fluorescence microscope (Nikon Eclipse Ti-S, CCD: Ri1) and then under a laser scanning confocal microscope (Leica TCS sp5, Germany) and were used for confocal luminescence imaging with a 63 × oil immersion objective lens.

### Cytotoxicity of GNRs@MSNs@PCM@miR-197 to Prostate Cancer Stem Cell Under Near-Infrared Laser Irradiation

Prostate cancer stem cells at the logarithmic growth phase were inoculated onto 96-well plates at a density of 5,000 cells/well, 1 day before the experiment, and the culture medium was discarded after 24 h of being inside the cell incubator, with different concentrations of AbCD133@GNRs@MSNs. The cells were cultured at 37°C for 4 h and then irradiated with a near-infrared laser (808 nm, 3 W/cm^2^). After 24 or 48 h of incubation, Cell Counting Kit-8 (CCK-8) assay was used to detect cell viability.

### *In vivo* Antitumor Efficacy of Prostate Cancer Stem Cell

Eight-week-old nude mice with an average weight of 18 g were used in this study. Each mouse was injected with 40 μl of the anesthetic before operation. A PCSC suspension was mixed with Matrigel at a ratio of 1:1. Each mouse was injected with 1 × 10^6^ cells, and the total volume of the injection was 50 μl. The mice were reared under good laboratory conditions (temperature 25 ± 2°C; relative humidity 50 ± 20%) with dark and light cycles (12/12 h) and access to standard balanced diet. The mice were observed every day after operation. After 2 weeks, the growth of the solid tumors was observed; and the length, width, and height were measured using a vernier caliper to calculate the volume of the tumor. After about 4 weeks, therapy was started when the tumor volume was 200 × 400 mm^3^. Mice were randomized into four groups of five mice per group. The groups of animals were treated variously: (1) control, (2) miR-197 inhibitor, (3) AbCD133@MSNs@AuNP, (4) AbCD133@MSNs@AuNP + 808 nm laser exposure 10 min, (5) AbCD133@MSNs@AuNP@miR-197 inhibitor, and (6) AbCD133@MSNs@AuNP@miR-197 inhibitor + 808 nm laser exposure 10 min. Treatment was performed three times a week, and tumor growth was measured every 5 days, while the tumor volume was also calculated.

### Statistical Analysis

SPSS 16.0 statistical software (IBM Corporation, Armonk, NY, United States) was used to analyze the data. Non-paired *t* test was used to estimate statistical differences between two groups, while one-way analysis of variance (ANOVA) was applied to determine differences among three or more groups. A *P* value of < 0.05 was considered to indicate a statistically significant difference.

## Results

### MiR-197 Is Overexpressed in Prostate Cancer Tissues and Prostate Cancer Cells

**FIGURE 1:**
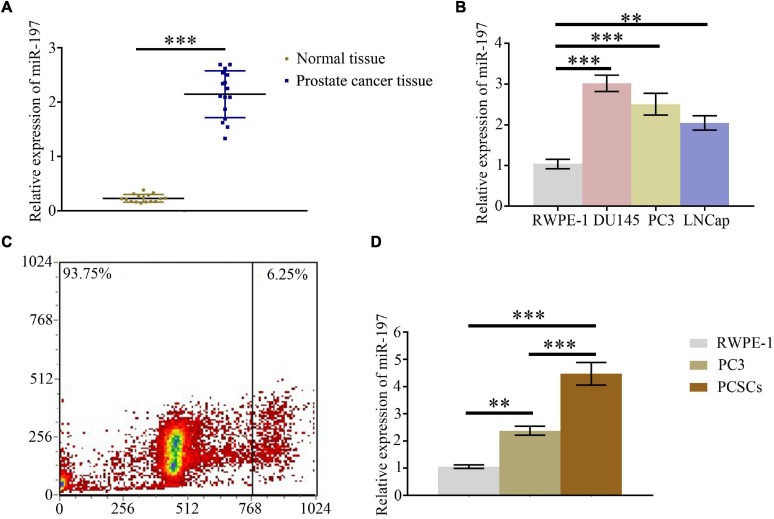
MiR-197 is overexpressed in prostate cancer tissues and prostate cancer cells. **(A)** qPCR assay was used to detect the expression of miR-197 in prostate cancer tissues and different prostate cancer cells. **(B)** qPCR assay was used to determine the expression of miR-197 in prostate cancer cells lines (RWPE-1, DU145, PC3, and LNCaP). **(C)** CD133 overexpressing PC3 cells were selected using flow cytometry. **(D)** qPCR assay was used to detect the expression of miR-197 in RWPE-1, PC3, and prostate cancer stem cells (PCSCs). *p < 0.05, **p < 0.01, ***p < 0.001.

qPCR assay was conducted to detect the expression level of miR-197 in PC tissues and different PC cells. The results indicated that miR-197 was significantly overexpressed in PC tissues (Figure 1A), compared with paired normal tissue, and that the expression of miR-197 in the PC cells lines (DU145, PC3, and LNCaP) was significantly increased than in the normal prostate cell line, RWPE-1 (Figure 1B). To study miR-197 expression in PCSCs, CD133-overexpressing PC3 cells were selected using flow cytometry (Figure 1C), and then miR-197 expression was detected in the RWPE-1, PC3, and PCSCs. The results showed that miR-197 expression in the PCSCs was significantly higher than in RWPE-1 and PC3 cells (Figure 1D).

### MiR-197 Regulates ITGAV Expression in the Prostate Cancer Tissues and Prostate Cancer Cells

**FIGURE 2:**
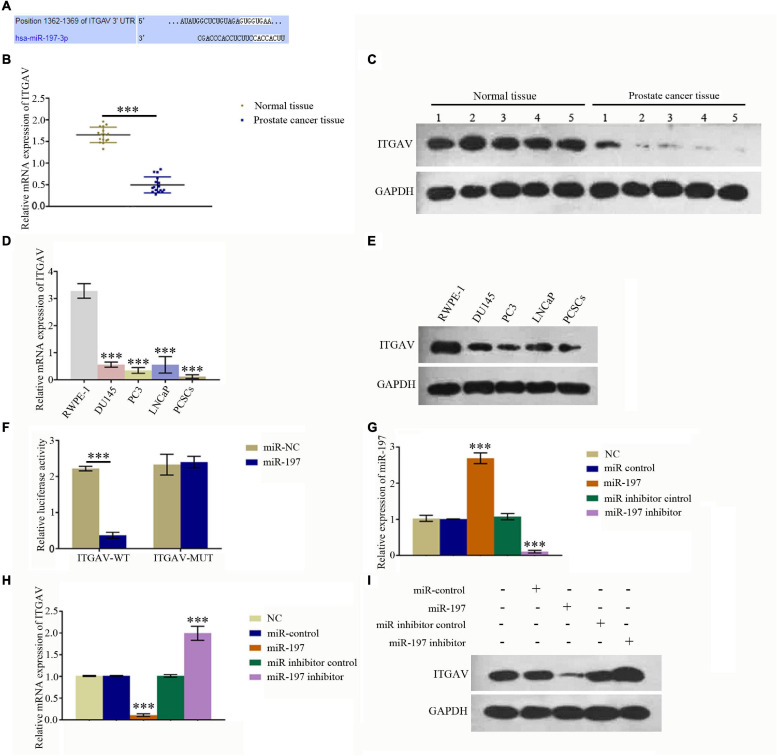
MiR-197 regulated ITGAV expression in prostate cancer tissues and cells. **(A)** An online bioinformatics analysis was conducted using the TargetScan 7.2 database to determine whether ITGAV could be regulated by miR-197 and 1362–1369 of ITGAV 3′-UTR was found to target miR-197. **(B)** qRT-PCR assay showed the expression of ITGAV mRNA was lower in prostate cancer tissues. **(C)** Western blotting assay showed the expression of ITGAV protein was lower in prostate cancer tissues. **(D)** qRT-PCR assay found the expression of ITGAV mRNA was lowest in prostate cancer stem cells (PCSCs). **(E)** Western blotting assay determined the protein expression levels of ITGAV was lower in PC3 and PCSCs. **(F)** Dual luciferase reporter assay confirmed the direct interaction between miR-197 and ITGAV mRNA. **(G)** qRT-PCR assay determined the expression of miR-197 in PCSCs cells after transfection with the miR control, miR-197 mimic, miR inhibitor control and miR-197 inhibitor. The results showed the expression of miR-197 was lowest in miR-197 inhibitor group. **(H)** qRT-PCR assay determined the expression of ITGAV mRNA in PCSCs cells after transfection with the miR control, miR-197 mimic, miR inhibitor control and miR-197 inhibitor. The results showed the expression of ITGAV mRNA was highest in miR-197 inhibitor group. **(I)** Western blotting assays was used to detect the expression of ITGAV, CD133 and the phosphorylation of STAT5 and ERK in PCSCs after transfection with the miR control, miR-197 mimic, miR inhibitor control and miR-197 inhibitor. *p < 0.05, **p < 0.01, ***p < 0.001.

An online bioinformatics analysis was conducted using the TargetScan 7.2 database to predict proteins that may be regulated by miR-197. The results showed that 1,362–1,369 of ITGAV 3′-UTR might be the target of miR-197 (Figure 2A). qPCR and western blotting assays were conducted to detect the expression of ITGAV in PC tissues and different PC cells. The results showed that mRNA and protein expression levels of ITGAV were significantly decreased in PC tissues, compared with normal tissues (Figures 2B,C), and that mRNA and protein expression levels of ITGAV in PC cells (DU145, PC3, and LNCaP) as well as PCSC were also significantly decreased, compared with normal prostate cells (RWPE-1). Dual-luciferase reporter assay was conducted to study direct interactions between miR-197 and ITGAV mRNA, and the relative luciferase value was found to have decreased significantly after miR-197 transfection in the ITGAV wild-type (WT) group. However, the relative luciferase value did not show a significant change in the ITGAV mutant group (Figure 2F). Meanwhile, the miR-197 mimic could significantly inhibit ITGAV and CD133 expression and significantly decrease the phosphorylation of STAT5 and ERK, while the miR-197 inhibitor produced an opposite effect (Figures 2G–I). These results indicated that miR-197 directly regulates ITGAV/STAT5 pathway in PC tissues and PC cells and that miR-197 plays an important role in PC development.

### MiR-197 Inhibitor Functions as a Potential Suppressor of Prostate Cancer

**FIGURE 3:**
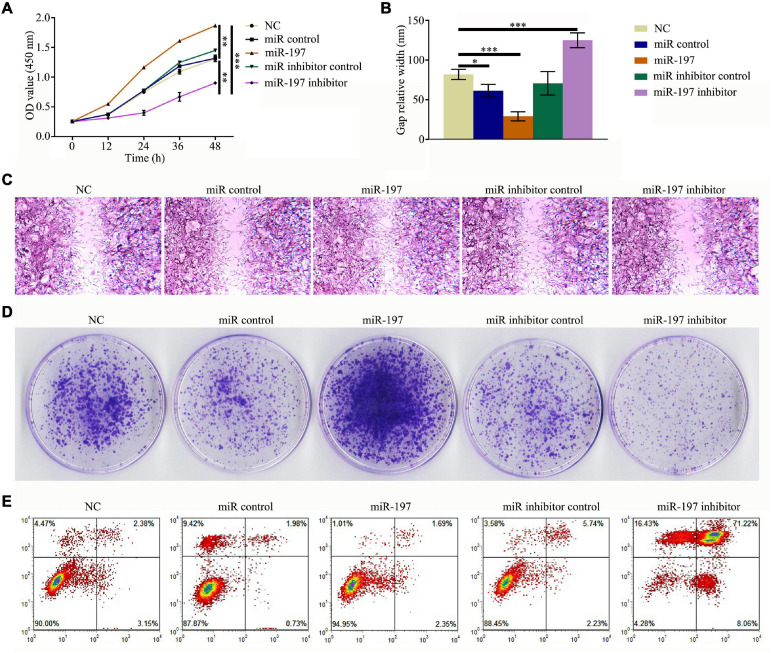
MiR-197 inhibitor is a potential suppressor of prostate cancer. PC3 was transfected with miR-197 mimics or miR-197 inhibitor. **(A)** Cell Counting Kit-8 (CCK-8) assay was conducted to detect the level of cell proliferation. **(B,C)** Cell scratch assay was conducted to determine the effect of miRNAs on prostate cancer stem cell (PCSC) migration. **(D)** Colony formation assay was conducted to observe PCSC growth features. **(E)** An Annexin V-FITC/PI apoptosis detection kit and flow cytometry were used to evaluate PCSC apoptosis. *p < 0.05, **p < 0.01, ***p < 0.001.

To study the effect of miR-197 on the development of PC, PCSCs were transfected with either a miR-197 mimic or a miR-197 inhibitor. Then, CCK-8 assay was used to detect cell proliferation, cell scratch assay was used to test the effect of the miRNA on PCSC migration, colony formation assay was used to observe cell growth features, and an Annexin V-FITC/PI apoptosis detection kit and flow cytometry were used to determine cell apoptosis. The results showed that the miR-197 inhibitor could significantly decrease the proliferation and migration levels of the PCSCs as well as significantly decrease the level of PCSC apoptosis (Figures 3A–E). Therefore, these results indicate that the miR-197 inhibitor is a potential suppressor of PC development and progression.

### Synthesis and Characterization of Mesoporous Silica-Coated Gold Nanorods

**FIGURE 4:**
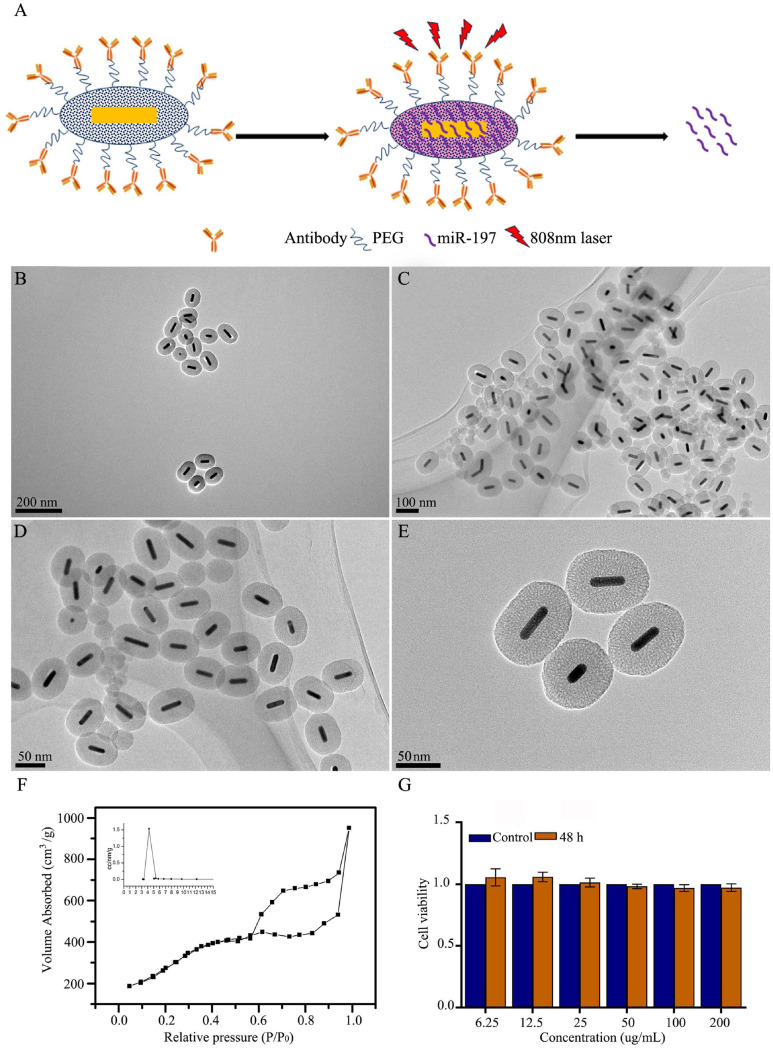
Synthesis and characterization of mesoporous silica-coated gold nanorods. **(A)** GNR@MSNs and AbCD133@GNR@MSNs synthesis. **(B–E)** transmission electron microscopy (TEM) image of GNR@MSNs. **(F)** The mesoporous morphology and mesoporous distribution (inset) of the GNR@MSNs were determined using N2 adsorption–desorption isotherms. **(G)** Cell Counting Kit-8 (CCK-8) assay was conducted to test the biocompatibility of AbCD133@GNR@MSNs on prostate cancer stem cells (PCSCs).

It is necessary to develop nanocarriers with good biocompatibility, loading capacity, cell internalization, and release performance for use with miRNA-based methods of cancer treatment. In this study, we first synthesized mesoporous silica-coated GNRs (GNR@MSNs) and then modified the CD133 antibodies on the surface of the GNR@MSNs to synthesize AbCD133@GNR@MSNs (Figure 4A). Mesoporous silica provides a high drug loading capacity and good release performance, while GNR nanorods contribute photothermal properties. The AbCD133 in AbCD133@GNR@MSNs can target PCSCs, which are a small group of prostate tumor cells that determine the proliferation, invasion, metastasis, and drug resistance of PC. Figures 4B–E provide a TEM image of GNR@MSNs, while Figure 4F shows the mesoporous morphology and pore size distribution of the GNR@MSNs. Then, CCK-8 assay was conducted to test the biocompatibility of AbCD133@GNR@MSNs to PCSCs. The result showed that a significant cytotoxicity effect was not produced when the concentration was between 6.25 and 200 μg/ml (Figure 4G).

### MicroRNA Loading Capacity, Release Performance, Photothermal Properties, and Cell Internalization of AbCD133@GNR@MSNs

**FIGURE 5:**
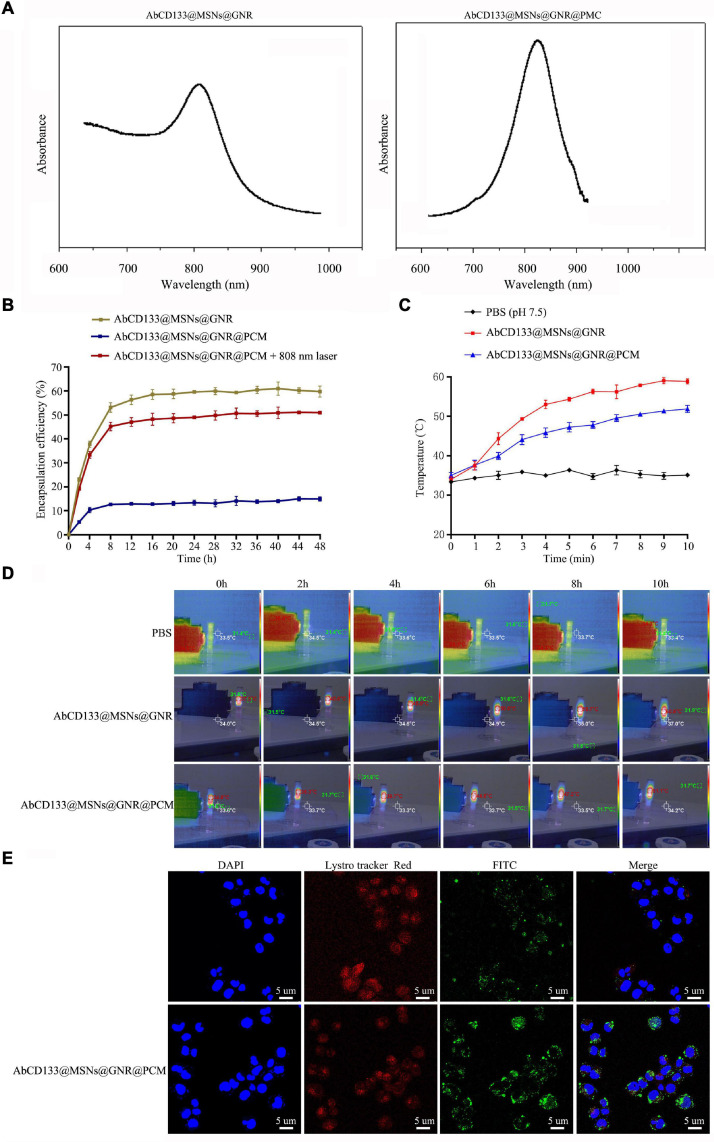
MicroRNA loading capacity, release performance, photothermal properties, and cell internalization of AbCD133@GNR@MSNs. **(A)** UV absorption spectrum detection of AbCD133@GNR@MSNs and AbCD133@GNR@MSNs@PCM. **(B)** Cell Counting Kit-8 (CCK-8) assay was used to test the biocompatibility of AbCD133@GNR@MSNs with or without 808-nm laser irradiation. **(C,D)** The photothermal properties of AbCD133@GNR@MSNs and AbCD133@GNR@MSNs@PCM determined under 808-nm laser irradiation. **(E)** Laser confocal microscopy was used to detect prostate cancer stem cell (PCSC) internalization of GNR@MSNs and AbCD133@GNR@MSNs.

To achieve controllable photothermal release, PCMs were used to seal the mesoporous AbCD133@GNR@MSNs. PCM can be welded under 808-nm near-infrared laser irradiation, and AbCD133@GNR@MSNs will slowly release the miR-197 inhibitor. NanoDrop 3000 was used to detect changes in miR-197 inhibitor concentration before and after loading. The results showed that 1 mg of AbCD133@GNR@MSNs could load 35.42 μg of the miR-197 inhibitor. Subsequently, a semi-permeable membrane release detection model and NanoDrop 3000 were used to study the AbCD133@GNR@MSNs release properties of the miR-197 inhibitor with or without 808-nm laser irradiation. According to the result shown in Figure 5A, both AbCD133@GNR@MSNs and AbCD133@GNR@MSNs@PCM have a maximum absorption peak at about 808 nm. The results showed almost no miR-197 inhibitor release under 808-nm laser irradiation when the AbCD133@GNR@MSNs were sealed with PCM, while the AbCD133@GNR@MSNs without PCM showed a release performance that was similar to that of AbCD133@GNR@MSNs@PCM under 808-nm laser irradiation. The results showed that there was a rapid release of the miR-197 inhibitor at 0–8 h, and then the miR-197 inhibitor could be released stably to produce a long-term effect (Figure 5B). Figures 5C,D show the photothermal properties of the AbCD133@GNR@MSNs. According to the results, the temperature of both the AbCD133@GNR@MSNs and AbCD133@GNR@MSNs@PCM can reach above 40°C after 3 min of 808-nm laser irradiation and can continue to increase up to 60°C within 10 min. Specifically killing CSCs using targeted drug carriers is a novel strategy used in cancer treatment. We modified the antibody of the PCSC marker, AbCD133, on the surface of the GNR@MSNs and then constructed FITC-labeled AbCD133@GNR@MSNs, while laser confocal microscopy was used to detect PCSC internalization of the AbCD133@GNR@MSNs. The results showed that CD133 antibody modification could significantly improve PCSC internalization of the GNR@MSNs. A significant increase of FITC-labeled AbCD133@GNR@MSNs (green fluorescence), compared with FITC-labeled GNR@MSNs, was observed in PCSCs (Figure 5E).

### The Effect of AbCD133@GNR@MSNs@miR-197 Inhibitor on the Development of Prostate Cancer Stem Cells Under 808-nm Laser Irradiation

**FIGURE 6:**
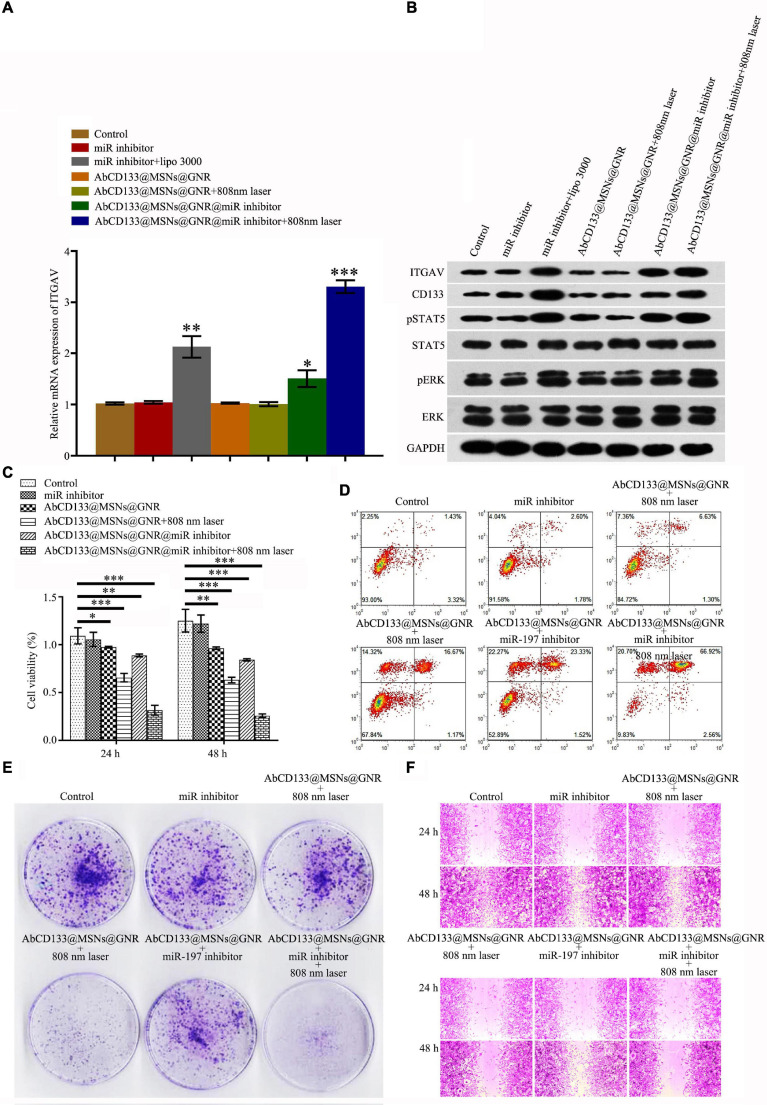
The effect of AbCD133@GNR@MSNs@miR-197 inhibitor on the development of PC3 prostate cancer cells under 808-nm laser irradiation. **(A)** qPCR assay was used to test the mRNA expression of ITGAV. **(B)** Western blotting assay was used to test the expression of ITGAV and CD133 and the phosphorylation of STAT5, ERK, and ITGAV-related proteins. **(C)** Cell Counting Kit-8 (CCK-8) assay was used to test the cell viability of the prostate cancer stem cells (PCSCs). **(D)** An Annexin V-FITC/PI apoptosis detection kit was used to detect PCSC apoptosis. **(E,F)** Colony formation experiment and cell scratch assay were conducted to test the cell invasion and infection abilities of the PCSCs. *p < 0.05, **p < 0.01, ***p < 0.001.

To test the effect of the AbCD133@GNR@MSNs@miR-197 inhibitor on the ITGAV relative pathway, qPCR and western blotting assay were used to test the expression of ITGAV and ITGAV-related proteins, and results showed that the AbCD133@GNR@MSNs@miR-197 inhibitor with 808-nm laser irradiation could significantly increase the expression of ITGAV and CD133 and significantly increase the phosphorylation of STAT5 and ERK (Figures 6A,B). To test the effect of the AbCD133@GNR@MSNs@miR-197 inhibitor on the development of PCSCs, CCK-8 assay was conducted to determine cell viability, and the result showed that after irradiation with an 808-nm laser, the AbCD133@GNR@MSNs@miR-197 inhibitor could significantly decrease the cell viability of the PCSCs (Figure 6C). An Annexin V-FITC/PI apoptosis detection kit was used to determine cell apoptosis, and the results showed that the AbCD133@GNR@MSNs@miR-197 inhibitor with 808-nm laser irradiation could significantly induce PCSC apoptosis (Figure 6D). Then, clone formation experiment and cell scratch assay were conducted to determine the cell invasion and infection abilities. According to the results, the AbCD133@GNR@MSNs@miR-197 inhibitor with 808-nm laser irradiation could significantly decrease the clone formation rate (Figure 6E) and significantly inhibit the scratch gap growth rate (Figure 6F).

### The Effect of AbCD133@GNR@MSNs@miR-197 Inhibitor on the Prostate Cancer Stem Cells in the Solid Tumor Model Under 808-nm Laser Irradiation

**FIGURE 7:**
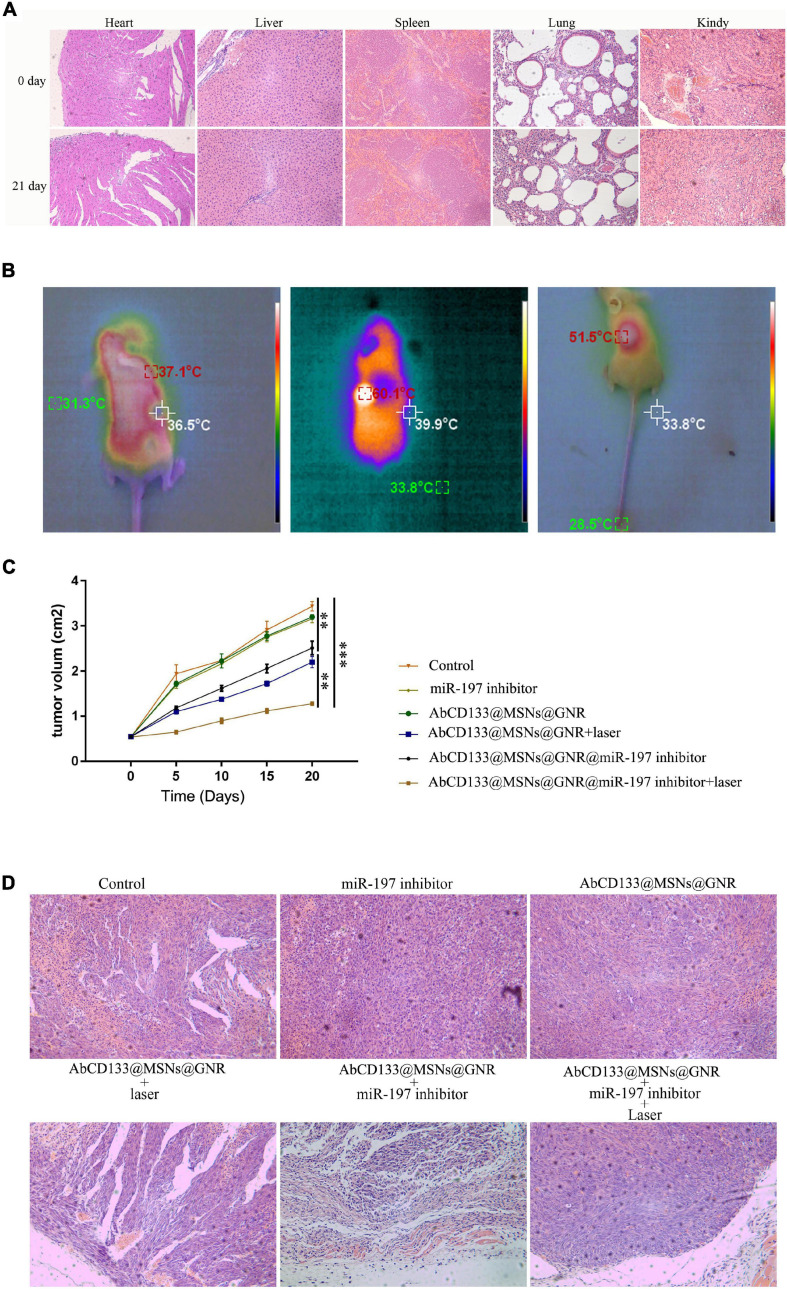
The effect of AbCD133@GNR@MSNs@miR-197 inhibitor on prostate cancer stem cells (PCSCs) in a solid tumor model under 808-nm laser irradiation. **(A)** The acute toxicity of AbCD133@GNR@MSNs on the mice was determined using H&E staining assay to observe the effect of AbCD133@GNR@MSNs on the heart, liver, spleen, lung, and kidneys of the mice. **(B)** AbCD133@GNR@MSNs@miR-197 inhibitor could increase the temperature of the PCSCs in the solid tumors under 808-nm laser irradiation. **(C)** AbCD133@GNR@MSNs@miR-197 inhibitor could significantly inhibit the growth of the PCSCs in the solid tumors. **(D)** H&E staining assay was conducted to observe the effect of AbCD133@GNR@MSNs on the PCSCs in solid tumors under 808-nm laser irradiation. *p < 0.05, **p < 0.01, ***p < 0.001.

We explored the effect of the AbCD133@GNR@MSNs@miR-197 inhibitor on the PCSCs in solid tumor formation and growth under 808-nm laser irradiation. Firstly, the acute toxicity of AbCD133@GNR@MSNs on mice was evaluated, and the results showed that AbCD133@GNR@MSNs have no effect on the weight increase of the mice, and H&E staining assay was used to observe the effect of AbCD133@GNR@MSNs on the heart, liver, spleen, lung, and kidneys of the mice (Figure 7A). Then, the effect of the AbCD133@GNR@MSNs@miR-197 inhibitor on the PCSC solid tumor growth was evaluated. Figure 7B shows that the AbCD133@GNR@MSNs@miR-197 inhibitor could increase the temperature of the PCSCs in solid tumors with 808-nm laser irradiation to significantly inhibit the growth of the PCSC solid tumors (Figure 7C). Then, H&E staining assay was conducted to observe the effect of AbCD133@GNR@MSNs on PC3 PC cell solid tumors under 808-nm laser irradiation. The results showed that the destructive effect of the AbCD133@GNR@MSNs@miR-197 inhibitor under 808-nm laser irradiation was significantly higher on tumor tissue destruction than cells of the other groups (Figure 7D).

## Discussion

Prostate cancer is one of the most significant malignant tumors that affects males and has the second highest mortality rate of all tumors. The diagnosis and treatment of PC have become an important research focus of modern medicine (Mabuchi et al., 2020; Steiner et al., 2020). Current treatments for PC can be divided into endocrine therapy [bilateral orchiectomy, luteinizing hormone-releasing hormone (LHRH) analog treatment, anti-androgen therapy, androgen combined blocking therapy, and intermittent anti-androgen therapy], surgical treatment (radical prostatectomy and intracavitary surgery for adenocarcinoma), radiotherapy, and chemotherapy. However, the traditional methods of treatment for PC produce obvious side effects and a high recurrence rate, which seriously affect the treatment effect and the quality of life of patients. PCSCs are a small number of cells that drive the occurrence and development of tumor. They play an important role in the development, self-renewal, and drug resistance of PC (Gratwohl et al., 2002; Hermann et al., 2007; Miki et al., 2007; O’Connor et al., 2014; Zhang et al., 2021), Research and development of drugs targeting PCSCs is one of the important ways to treat PC.

In recent years, the importance of gene therapy has been widely recognized in PC research due to it highly targeted effects and fewer side effects. Gene therapy can inhibit the growth of PC cells by regulating the protein expression of targeted genes, and PC therapy based on miRNAs is a research hotspot in this field (Li N. et al., 2020; Yue et al., 2020; An et al., 2021). MiRNAs can bind to the 3′ untranslated region of the targeted gene mRNA and can regulate the expression of downstream target genes. In recent years, many miRNAs associated with PC have been identified and have been confirmed to be closely associated with the occurrence and development of PC (Hou et al., 2020; Chen et al., 2021; Wang et al., 2021). Previous studies have reported that miR-197 is significantly overexpressed in the serum of PC patients, which indicates that miR-197 plays an important role in the occurrence and development of PC. However, the molecular mechanism of miR-197 in the bone metastasis of PC has not as yet been reported (Yuan et al., 2019).

In this study, we first studied the molecular mechanism by which miR-197 affects the development of PC. Our study verified that miR-197 is significantly overexpressed in the PC tissues and PC cells, compared with normal PC cells. These results indicate that miR-197 may play an important role in the proliferation, invasion, and metastasis of PC cells. Then, we found and confirmed that miR-197 can regulate ITGAV expression of PC cells by directly binding to the 3′-UTR of ITGAV. Previous studies have reported that ITGAV can regulate the proliferation, invasion, and metastasis of several cancers, such as gastric cancer, breast cancer, esophageal cancer, and colorectal cancer (Waisberg et al., 2014; Ma et al., 2016; Wang et al., 2019; Cheuk et al., 2020; Li Q. et al., 2020). Our study showed that miR-197 affects the proliferation, invasion, and metastasis of PC cells by regulating ITGAV expression. Together, these results indicated that the miR-197 inhibitor may potentially suppress the development and progression of PC, which can be used for the treatment of PC.

For potential clinical application, many miR-197 inhibitors need to be introduced into PC cells, and they must avoid degradation caused by genetic drugs during and after entering cancer cells. In addition, genetic drugs should be able to release a stable drug concentration after entering cancer cells. In this study, we synthesized an AbCD133@GNR@MSNs@miR-197 inhibitor drug carrier. The mesoporous structure of the MSNs could load many miR-197 inhibitors (35.42 μg of miR-197 inhibitor/mg AbCD133@GNR@MSNs), and the miR-197 inhibitor showed good release performance after entering the cells. The modified CD133 antibodies on the surface of the nano drug carriers can specifically enrich drug carriers around the PCSCs and results in an increased quantity of drugs entering PCSCs. This increase in quantity leads to a more effective effect by selectively targeting and suppressing CSCs. GNR, the core of the drug carrier, not only can release heat to kill cancer cells after being exposed to near-infrared radiation but also can melt the PCM used to seal mesoporous structure to achieve photothermal controlled release of the miR-197 inhibitor. The pharmacodynamic effect of the AbCD133@GNR@MSNs@miR-197 inhibitor on PCSCs *in vivo* and *in vitro* under near-infrared radiation was studied. The results showed that the AbCD133@GNR@MSNs@miR-197 inhibitor could significantly decrease cell viability of the PCSCs, induce significant PCSCs apoptosis, and significantly decrease the clone formation rate and invasion ability of the PCSCs. The AbCD133@GNR@MSNs@miR-197 inhibitor could also significantly inhibit the growth of PCSCs in solid tumors under 808-nm laser irradiation.

## Conclusion

In summary, our study found that miR-197 affected the proliferation, invasion, and metastasis of PC cells by regulating ITGAV expression. The AbCD133@GNR@MSNs@miR-197 inhibitor prepared in this study could suppress the development and growth of PCSCs *in vitro* and in solid tumors not only by targeting the ITGAV but also through photothermal therapy. Our study not only provides a theoretical basis and for the clinical treatment of PC but also provides a research scheme of drug loading and miRNA-based photothermal controlled therapy for prostate cancer.

## Data Availability Statement

The original contributions presented in the study are included in the article/supplementary material, further inquiries can be directed to the corresponding author/s.

## Ethics Statement

The experiments animals were performed under approval of the Institutional Animal Care and Use Committee of Changzheng Hospital, Naval Medical University, Shanghai, China.

## Author Contributions

GJ designed research and performed the experiments. YZ and TD performed the experiments. WC and JL interpreted results of the experiments. GJ and CL analyzed data and prepared figures. DX and ZW designed the experiments, provided the experimental insight, and edited the manuscript. All authors read and approved the final manuscript.

## Conflict of Interest

The authors declare that the research was conducted in the absence of any commercial or financial relationships that could be construed as a potential conflict of interest.
